# Introduction of a Multidisciplinary Preoperative Clinic at Juntendo University Hospital - A Retrospective Observational Study Focusing on Effects of Preoperative Interventions on Clinical Outcomes

**DOI:** 10.14789/jmj.JMJ23-0023-OA

**Published:** 2023-09-29

**Authors:** YUKI UMENO, SEIJI ISHIKAWA, OSAMU KUDOH, SHUKO NOJIRI, GAUTAM DESHPANDE, EIICHI INADA, MASAKAZU HAYASHIDA

**Affiliations:** 1 Department of Anesthesiology and Pain Medicine, Graduate School of Medicine, Juntendo University, Tokyo, Japan; 1 Department of Anesthesiology and Pain Medicine, Graduate School of Medicine, Juntendo University, Tokyo, Japan; 2 Department of Anesthesiology and Pain Medicine, Faculty of Medicine, Juntendo University, Tokyo, Japan; 2 Department of Anesthesiology and Pain Medicine, Faculty of Medicine, Juntendo University, Tokyo, Japan; 3 Medical Technology Innovation Center, Juntendo University, Tokyo, Japan; 3 Medical Technology Innovation Center, Juntendo University, Tokyo, Japan; 4 Department of General Medicine, Juntendo University Hospital, Tokyo, Japan; 4 Department of General Medicine, Juntendo University Hospital, Tokyo, Japan

**Keywords:** postoperative complication, preoperative clinic, preoperative evaluation, preoperative management

## Abstract

**Objectives:**

To investigate the effects of interventions provided by a multidisciplinary team consisting of anesthesiologists, dentists, pharmacists, and nurses at a Preoperative Clinic (POC) on postoperative outcomes.

**Methods:**

We retrospectively investigated patients who underwent preoperative evaluation at the POC at Juntendo University Hospital between May and July, 2019. Patients were divided into intervention and non-intervention groups according to whether they received intervention(s) at the POC or not. Postoperative outcomes were compared between the groups, before and after propensity score (PS) matching.

**Results:**

We investigated 909 patients who completed POC evaluation and underwent surgery. Patients in the intervention group (n = 455 [50.1%]) received at least one intervention delivered, in the order of higher delivery frequencies, by dentists, pharmacists, nurses, and anesthesiologists. Before PS matching, the intervention group was associated with older age, more frequent cardiovascular comorbidities, and higher ASA-PS grades than the non-intervention group, while neither frequencies nor severities of postoperative complications differed between the groups. These outcomes did not differ between 382 PS-matched pairs with comparable risk factors either.

**Conclusions:**

Before PS matching, postoperative outcomes did not differ between the groups, although the intervention group was associated with higher risks. These suggested that POC interventions could have improved postoperative outcomes in the higher-risk intervention group to the same level as in the non-intervention group. However, such potential beneficial effects of interventions could not be proven after PS matching. Further studies are required to elucidate effects of POC interventions on postoperative outcomes.

## Introduction

Robust preoperative assessment and appropriate management of surgical patients by anesthesiologists are essential to optimize postoperative outcomes^1)^. Nonetheless, preoperative assessment by anesthesiologists has conventionally been carried out during a limited time after patients' hospitalization^2)^. As such an approach may result in suboptimal outcomes, preoperative patient management at an outpatient-based preoperative clinic (POC) has been increasing^3)^. Currently, however, there is no standardized structure or guideline for a POC system.

Organizational designs of the POC can roughly be divided into single-professional and multi- professional models^4)^. Various studies regarding single-professional models have been reported^5-11)^. However, studies of multi-professional models remain limited, and the benefits of preoperative evaluation provided by multi-professional POC teams remain to be clarified.

This study aimed to introduce a multidisciplinary POC team at our institute, consisting of anesthesiologists, dentists or dental hygienists, pharmacists, and nurses, and to assess the impact of interventions provided by the POC team on clinical outcomes.

## Materials and Methods

The Research Ethics Committee at Juntendo University Hospital (JUH) approved this study (No. H19-0157, September 20th, 2019) with a waiver of informed consent. This study investigated a single-center historical cohort of patients who visited the POC and underwent surgery at JUH, in accordance with the Strengthening the Reporting of Observational Studies in Epidemiology (STROBE) guidelines^12)^.

### Conceptual framework of the POC at JUH

Approximately 10,000 surgeries under anesthesia managed by anesthesiologists are performed annually at JUH. Since the establishment of the POC in 2019, preoperative assessment and evaluation of non-obstetric patients scheduled for elective surgery have been carried out at the POC. The POC is managed by a collaborative team consisting of above-mentioned four disciplines. In principle, a patient has interviews and/or consultations with a pharmacist, dentist or dental hygienist, anesthesiologist, and nurse in this order. While the order may vary depending on provider availability, an interview with a pharmacist and oral screening by a dental staff are completed before a consultation with an anesthesiologist, as the anesthesiologist requires relevant information on medication and oral health for anesthetic planning. An interview with a nurse is typically performed last as it also involves confirmation of written informed consent.

One of the primary roles of pharmacists is to manage patients' medication, such as anti-coagulants, in preparation for surgery. The dental staff perform oral health screening, and clearly indicate the condition of each tooth by color/shape-coding in the medical record. This practice enables anesthesiologists and nurses in the operating room to recognize any tooth requiring caution during intubation and to compare pre- and post-operative oral conditions. The roles of the anesthesiologists at the POC, such as evaluating preoperative health conditions and providing information on anesthesia, remain unchanged from those previously performed after patients' hospitalization. However, the POC provides opportunities for patients to receive information on anesthesia in advance to have sufficient time, allowing for better degrees of comprehension of, and self-preparation for, scheduled procedures. To promote patient comprehension further, patients are encouraged to watch original movies showing typical anesthesia procedures for scheduled surgery during their waiting time. Nurses' roles include arranging patients' orders, explaining an in-hospital perioperative flow of care, assessing physical conditions such as skin and joint health, confirming completion of required documentations, and informing a relevant section staff of patient information. Having nurses be the final discipline to interact with patients also allows patients to ask any open questions. Information on preoperative evaluation is shared among the multidisciplinary team using check sheets and POC medical records.

Preoperative interventions in this study refer to any intervention delivered by any POC discipline. These may include anesthesiologists ordering additional examinations based on their POC assessment, dentists/dental hygienists ordering oral treatment from the Department of Oral and Maxillofacial Surgery, and any involved disciplines consulting with specialists for expert advice. Among diverse roles of nurses, however, nurse-delivered interventions in this study were limited to interactions with other disciplines/sections, considering eligible quantitative data collection. Interventions are performed when each profession deemed them necessary. However, necessary interventions are not always performed, since some patients are unwilling to receive them, e.g. because of the extra time and/or expense required to perform them. In addition, a decision as to whether or not a certain intervention is necessary for a certain health condition can vary among individuals in each profession because there are not always strict criteria for interventions to be performed by the profession. Therefore, in the present study, it was highly likely that not all patients in the non-intervention group did not need interventions, and conversely, not all patients in the intervention group needed interventions.

### Patient inclusion and data collection

**Figure 1:**
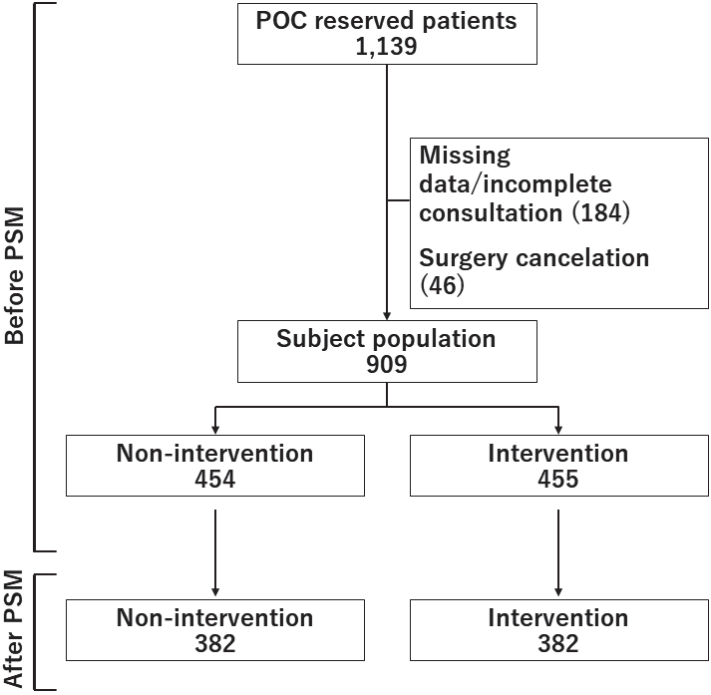
Flow chart outlining the inclusion and exclusion criteria of this study POC: Outpatient-based Preoperative Clinic PSM: Propensity score matching

Included were patients scheduled for elective surgery other than cardiovascular surgery and obstetric surgery, who visited the POC at JUH between May 7th and July 31st, 2019. Patients scheduled for obstetric surgery were not included because their preoperative conditions are specifically evaluated by our obstetric anesthesia team. Further, patients scheduled for cardiovascular surgery were not included because initially, they did not visit the POC during the study period, although currently, they visit the POC. Excluded were patients whose data were insufficient and those whose surgeries were canceled subsequently. Patients' demographic, anesthetic, and surgical data were collected, including age, sex, clinical departments, comorbidities, American Society of Anesthesiologists physical status (ASA-PS), surgical procedures, and methods of anesthesia. Data on POC intervention(s) were collected from POC medical records. Patients receiving any intervention and those receiving no intervention were grouped into, and defined as, the intervention group and the non-intervention group, respectively (Figure 1).

Postoperative complications included hospital mortality, reoperations, and any deviation from the natural postoperative course^13)^, including postoperative nausea and vomiting (PONV), delirium, any intubation-related airway injuries, and any organ dysfunction categorized into defined body parts^14)^. Severities of postoperative complications ranked by the Clavien-Dindo classification (CDC)^13)^ were also explored. While the CDC is typically classified into grades from I to V, we included patients without any complications as Grade 0. Further, we combined grades IVa and IVb into Grade IV because of difficult discrimination between single and multiple organ dysfunction (s). Information on the type and severity of postoperative complications was collected from the discharge summary as well as from other postoperative records in the electronic medical record.

### Statistical analysis

Continuous variables are summarized as Median [Interquartile Range]. Categorical variables are summarized as Number (Percentage). Data were compared between groups with the Mann-Whitney *U* test, Fisher's exact test, or the chi-square test. First, clinical backgrounds and postoperative outcomes were compared between intervention and non-intervention groups in the total cohort. Then, these were compared after the nearest neighbor propensity score (PS) matching in 1:1 ratio with a 0.05 caliper was applied to create PS-matched pairs of patients by adjusting differences in age, sex, clinical departments, surgical procedures, anesthesia methods, and ASA-PS grades, as reported in a previous study investigating the effect, on postoperative mortality, of a single-profession POC involving an intervention to encourage high-risk patients to visit the POC^6)^. To compare clinical backgrounds between the groups, a *p* value < 0.05 was considered statistically significant. Meanwhile, to compare two major endpoints, including frequencies of complications and severities of complications (ranked by CDC grades), a *p* value < 0.025 was considered statistically significant. Statistical analysis was performed using IBM SPSS Statistics 27.0.1.0. (IBM Corp., Armonk, NY, USA).

## Results

### Clinical backgrounds

During the study period, 1,139 patients who met the inclusion criteria visited the POC at JUH. After excluding 184 patients whose data were insufficient and 46 patients whose surgeries were canceled subsequently, 909 patients were included in the study (Figure 1). Among them, 455 patients (50.1%) and 454 patients (49.9%) were grouped into intervention and non-intervention groups, respectively (Figure 1, Table 1). Compared to the non-intervention group, the intervention group included higher proportions of the elderly (≥ 65 years; 211/455 [46.4%] vs. 148/454 [32.6%], *p* < 0.0001), females, patients with hypertension, patients with cardiovascular disease, and/or patients classed as ASA-PS grades ≥ 2 (338/455 [74.3%] *vs.* 283/454 [62.3%], *p* = 0.0001) (Table 1).

**Table 1:** Patients' demographics, surgical, and anesthetic characteristics in the total cohort, intervention group, and non-intervention group before and after propensity score (PS) matching

	Before PS matching					After PS matching			
	Total cohort *n* = 909	Intervention *n* = 455	Non-intervention *n* = 454	*p* value		Total cohort *n* = 764	Intervention *n* = 382	Non-intervention *n* = 382	*p* value
Age	58 [41-71]	63 [47-72]	52 [34-70]	<.0001		58 [44-71]	60.5 [45-71]	56 [41-71]	0.1530
Gender, females	475 (52.3)	255 (56.0)	220 (48.5)	0.0240		393 (51.4)	204 (53.4)	189 (49.5)	0.3109
Comorbidities^a)^	428 (47.1)	229 (50.3)	199 (43.8)	0.0540		370 (48.4)	183 (47.9)	187 (49.0)	0.8281
Hypertension	261 (28.7)	146 (32.1)	115 (25.3)	0.0278		223 (29.2)	113 (29.6)	110 (28.8)	0.8736
Respiratory	149 (16.4)	70 (15.4)	79 (17.4)	0.4214		138 (18.1)	67 (17.5)	71 (18.6)	0.7779
Cardiovascular	104 (11.4)	65 (14.3)	39 (8.6)	0.0089		77 (10.1)	39 (10.2)	38 (9.9)	1.0000
Diabetes mellitus	97 (10.7)	49 (10.8)	48 (10.6)	1.0000		82 (10.7)	38 (9.9)	44 (11.5)	0.5592
Neurological	53 (5.8)	32 (7.0)	21 (4.6)	0.1564		44 (5.8)	23 (6.0)	21 (5.5)	0.8768
ASA-PS									
1	288 (31.7)	117 (25.7)	171 (37.7)	0.0004		231 (30.2)	111 (29.1)	120 (31.4)	0.7641
2	566 (62.3)	305 (67.0)	261 (57.5)			487 (63.7)	247 (64.7)	240 (62.8)	
3	55 (6.1)	33 (7.3)	22 (4.8)			46 (6.0)	24 (6.3)	22 (5.8)	
Clinical departments				0.0003					0.2170
Orthopedic	262 (28.8)	144 (31.6)	118 (26.0)			220 (28.8)	115 (30.1)	105 (27.5)	
Urological	110 (12.1)	41 (9.0)	69 (15.2)			106 (13.9)	40 (10.5)	66 (17.3)	
Breast Oncologic	103 (11.3)	63 (13.8)	40 (8.8)			87 (11.4)	48 (12.6)	39 (10.2)	
Colorectal	72 (7.9)	38 (8.4)	34 (7.5)			58 (7.6)	29 (7.6)	29 (7.6)	
Esophageal & Gastric	63 (6.9)	34 (7.5)	29 (6.4)			50 (6.5)	30 (7.9)	20 (5.2)	
Pediatric	62 (6.8)	14 (3.1)	48 (10.6)			34 (4.5)	14 (3.7)	20 (5.2)	
Plastic & Reconstructive	61 (6.7)	29 (6.4)	32 (7.0)			51 (6.7)	24 (6.3)	27 (7.1)	
General Thoracic	50 (5.5)	27 (5.9)	23 (5.1)			47 (6.2)	26 (6.8)	21 (5.5)	
Hepatobiliary & Pancreatic	40 (4.4)	19 (4.2)	21 (4.6)			34 (4.5)	15 (3.9)	19 (5.0)	
Otorhinolaryngological	38 (4.2)	18 (4.0)	20 (4.4)			38 (5.0)	18 (4.7)	20 (5.2)	
Gynecological	25 (2.8)	13 (2.9)	12 (2.6)			19 (2.5)	11 (2.9)	8 (2.1)	
Neurosurgical	12 (1.3)	6 (1.3)	6 (1.3)			10 (1.3)	4 (1.0)	6 (1.6)	
Dermatological	3 (0.3)	2 (0.4)	1 (0.2)			1 (0.3)	1 (0.3)	1 (0.2)	
Ophthalmological	3 (0.3)	3 (0.7)	0 (0)			3 (0.3)	3 (0.8)	0 (0)	
Internal Medical	5 (0.5)	4 (0.9)	1 (0.2)			5 (0.7)	4 (1.0)	1 (0.3)	
Surgical procedures				0.2078					0.9021
Open	685 (75.4)	355 (78.0)	330 (72.7)			573 (75.0)	289 (75.7)	284 (74.3)	
Laparoscopic	187 (20.6)	81 (17.8)	106 (23.3)			154 (20.2)	74 (19.4)	80 (20.9)	
Endoscopic	21 (2.3)	10 (2.2)	11 (2.4)			21 (2.7)	10 (2.6)	11 (2.9)	
Robot-assisted	16 (1.8)	9 (2.0)	7 (1.5)			16 (2.1)	9 (2.4)	7 (1.8)	
Anesthesia methods				0.2042					1.0000
General anesthesia	886 (97.5)	440 (96.7)	446 (98.2)			749 (98.0)	374 (97.9)	375 (98.2)	
Other	23 (2.5)	15 (3.3)	8 (1.8)			15 (2.0)	8 (2.1)	7 (1.8)	
Regional anesthesia				0.6747					0.4850
None	486 (53.5)	238 (52.3)	248 (54.6)			425 (55.6)	206 (53.9)	219 (57.3)	
Epidural anesthesia	202 (22.2)	98 (21.5)	104 (22.9)			165 (21.6)	84 (22.0)	81 (21.2)	
Nerve blocks	126 (13.9)	71 (15.6)	55 (12.1)			96 (12.6)	56 (14.7)	40 (10.5)	
Spinal anesthesia	26 (2.9)	15 (3.3)	11 (2.4)			20 (2.6)	11 (2.9)	9 (2.4)	
Spinal-epidural	2 (0.2)	1 (0.2)	1 (0.2)			2 (0.3)	1 (0.3)	1 (0.3)	
Other	67 (7.4)	32 (7.0)	35 (7.7)			56 (7.3)	24 (6.3)	32 (8.4)	

Data are shown as Median [Interquartile Range] or Number (Percentage). Continuous variables were compared between Groups with the Mann-Whitney *U* test. Categorical variables were compared with the chi-square test or Fisher's exact test. a) Some patients had multiple comorbidities. ASA-PS, American Society of Anesthesiologists physical status.

### Interventions provided in the intervention group before PS matching

In 455 patients in the intervention group, interventions were delivered, in the order of higher delivery frequencies, by dentists/dental hygienists (*n* = 334 [73.4% of the intervention group]), pharmacists (*n* = 116 [25.5%]), nurses (*n* = 66 [14.5%]), and anesthesiologists (*n* = 59 [13.0%]) (Table 2).

**Table 2:** Numbers of preoperative interventions provided by multi-professions at the preoperative clinic (POC) in the intervention group before and after propensity score (PS) matching

Providers and interventions	Before PS matching (n = 455)	After PS matching (n = 382)
**Dentists/Dental hygienists**	**334 (73.4)**	**288 (75.4)**
Mouth guard preparation	190 (41.8)	159 (41.6)
Oral cleaning	139 (30.5)	130 (34.0)
Other	29 (6.4)	23 (7.6)
**Pharmacists**	**116 (25.5)**	**94 (24.6)**
**Medication instructions**	**90 (19.8)**	**74 (19.4)**
CAM	71 (15.6)	59 (18.1)
Anti-coagulant	11 (2.4)	7 (1.8)
Anti-rheumatic agent	8 (1.8)	1 (0.3)
Hormonal agent	2 (0.4)	2 (0.5)
Hypoglycemic agent	1 (0.2)	1 (0.3)
Anti-hypertensive agent	1 (0.2)	1 (0.3)
Other	3 (0.7)	3 (0.8)
**Consultations with specialists**	**39 (8.6)**	**29 (7.6)**
CAM	3 (0.7)	3 (0.8)
Anti-coagulant	15 (3.3)	9 (2.4)
Anti-rheumatic agent	8 (1.8)	6 (1.6)
Hormonal agents	3 (0.7)	3 (0.8)
Hypoglycemic agent	1 (0.2)	1 (0.3)
Anti-hypertensive agent	0 (0.0)	0 (0.0)
Other	9 (2.0)	7 (1.8)
**Nurses**	**66 (14.5)**	**49 (12.8)**
**Interactions with others**	**66 (14.5)**	**49 (12.8)**
**Anesthesiologists**	**59 (13.0)**	**43 (11.3)**
**Consultations with specialists**	**20 (4.4)**	**16 (4.2)**
**Additional examinations**	**41 (9.0)**	**29 (7.6)**
Venous ultrasound	15 (3.3)	10 (2.6)
Blood chemistry test (CMP)	6 (1.3)	6 (1.6)
Echocardiography	6 (1.3)	2 (0.5)
Blood coagulation test	3 (0.7)	2 (0.5)
D-dimer test	3 (0.7)	2 (0.5)
X-ray	3 (0.7)	2 (0.5)
Electrocardiogram	2 (0.4)	1 (0.3)
Pulmonary function test	1 (0.2)	1 (0.3)
Other	4 (0.9)	4 (1.0)

Data are shown as Number (Percentage). Some patients received multiple interventions. CAM, complementary and alternative medicine; CMP, comprehensive metabolic panel.

Following dental screening at the POC, 334 patients (73.4%) received care for any oral problems, especially for fragile teeth, at the Department of Oral and Maxillofacial Surgery, such as mouth guard preparation (*n* = 190 [41.8%]) and/or oral cleaning (*n* = 139 [30.5%]) (Table 2).

Pharmacists performed interventions in 116 patients (25.5%). Pharmacists instructed 90 patients (19.8%) to withhold or continue medications, such as complementary and alternative medicines (CAMs), including supplements and Chinese herbal medicines (*n* = 71 [15.6%]), and anti-coagulants (*n* = 11 [2.4%]) (Tables 2). In addition, pharmacists consulted with specialists for 39 patients (8.6%), e.g. regarding perioperative management of anti-coagulants (*n* = 15 [3.3%]) (Table 2).

Nurses performed interventions, which were limited in this study to interactions with other disciplines/sections, in 66 patients (14.5%), including informing other health care providers of patients' critical information (Table 2).

Anesthesiologists performed interventions in 59 patients (13.0%), including ordering additional examinations (*n* = 41 [9.0%]) and/or consulting with specialists for expert advice (*n* = 20 [4.4%]) (Table 2).

### Clinical outcomes

Neither frequencies of postoperative complications nor CDC grades indicating their severities differed between intervention and non-intervention groups (Table 3). Frequencies of intubation- related complications, mostly sore throats and/or hoarseness, did not differ between the two groups. No tooth injury occurred in either group.

By applying PS matching, 382 pairs of patients with comparable clinical backgrounds were created from both groups (Tables 1 & 3). Neither frequencies of postoperative complications nor CDC grades differed between the PS-matched groups (Table 3).

**Table 3:** Frequencies of postoperative complications and their severities according to Clavien-Dindo Classification (CDC) in the total cohort, intervention group, and non-intervention group before and after propensity score (PS) matching

	Before PS matching					After PS matching			
	Total cohort (*n* = 909)	Intervention (*n* = 455)	Non-intervention (*n* = 454)	*p* value		Total cohort (*n* = 764)	Intervention (*n* = 382)	Non-intervention (*n* = 382)	*p* value
Complications^a^^)^	272 (29.9)	143 (31.4)	129 (28.4)	0.3465		224 (29.3)	111 (29.1)	113 (29.6)	0.9367
Death	2 (0.2)	1 (0.2)	1 (0.2)	1.0000		1 (0.1)	0 (0.0)	1 (0.3)	1.0000
Reoperation	12 (1.3)	4 (0.9)	8 (1.8)	0.2634		8 (1.0)	2 (0.5)	6 (1.6)	0.2865
PONV	69 (7.6)	35 (7.7)	34 (7.5)	1.0000		55 (7.2)	30 (7.9)	25 (6.5)	0.5759
Intubation-related	55 (6.1)	26 (5.7)	29 (6.4)	0.6793		48 (6.3)	24 (6.3)	24 (6.3)	1.0000
Sore throat/hoarseness	50 (5.5)	24 (5.3)	26 (5.7)			43 (5.6)	22 (5.8)	22 (5.8)	
Lip injury	4 (0.4)	1 (0.2)	3 (0.7)			3 (0.4)	0 (0.0)	3 (0.8)	
Vocal cord paralysis	1 (0.1)	1 (0.2)	0 (0.0)			1 (0.1)	1 (0.3)	0 (0.0)	
Tooth injury	0 (0.0)	0 (0.0)	0 (0.0)			0 (0.0)	0 (0.0)	0 (0.0)	
Respiratory	37 (4.1)	22 (4.8)	15 (3.3)	0.3139		31 (4.1)	19 (5.0)	12 (3.1)	0.2711
Gastric	35 (3.9)	16 (3.5)	19 (4.2)	0.6105		31 (4.1)	14 (3.7)	17 (4.5)	0.7144
Cardiac	34 (3.7)	20 (4.4)	14 (3.1)	0.3824		27 (3.5)	13 (3.4)	14 (3.7)	1.0000
Infections	33 (3.6)	20 (4.4)	13 (2.9)	0.2873		26 (3.4)	15 (3.9)	11 (2.9)	0.5503
Delirium	28 (3.1)	19 (4.2)	9 (2.0)	0.0823		22 (2.9)	13 (3.4)	9 (2.4)	0.5173
Deep vein thrombosis	8 (0.9)	7 (1.5)	1 (0.2)	0.0691		3 (0.4)	2 (0.5)	1 (0.3)	1.0000
Other	90 (9.9)	45 (9.9)	45 (9.9)	1.0000		71 (9.3)	30 (7.9)	41 (10.7)	0.2125
CDC grade^b^^)^				0.2056					0.2035
0	637 (70.1)	312 (68.6)	325 (71.6)			540 (70.7)	271 (70.9)	269 (70.4)	
Ⅰ	157 (17.3)	78 (17.1)	79 (17.4)			130 (17.0)	62 (16.2)	68 (17.8)	
Ⅱ	84 (9.2)	49 (10.8)	35 (7.7)			69 (9.0)	37 (9.7)	32 (8.4)	
Ⅲa	15 (1.7)	11 (2.4)	4 (0.9)			14 (1.8)	10 (2.6)	4 (1.0)	
Ⅲb	10 (1.1)	3 (0.7)	7 (1.5)			7 (0.9)	2 (0.5)	5 (1.3)	
Ⅳ	4 (0.4)	1 (0.2)	3 (0.7)			3 (0.4)	0 (0.0)	3 (0.8)	
Ⅴ	2 (0.2)	1 (0.2)	1 (0.2)			1 (0.1)	0 (0.0)	1 (0.3)	

Data are shown as Number (Percentage). Groups were compared with the chi-square test or Fisher's exact test. a) Some patients developed multiple complications. b) CDC grades, including CDC grade 0 indicating no complication, were compared between the groups. PONV, postoperative nausea and vomiting.

## Discussion

Nearly half of preoperative patients visiting the multidisciplinary POC received at least one intervention. Interventions were delivered, in the order of higher delivery frequencies, by dentists/dental hygienists, pharmacists, nurses, and anesthesiologists. Initially, we intended to compare postoperative outcomes between patients who underwent surgery before and after the establishment of the POC to examine effects of the POC on outcomes, similar to our previous study investigating the effect of the POC system on the surgery cancellation rate^15)^. However, such a study design cannot be free from a time-dependent bias resulting from comparing data collected in entirely different study periods. Further, what could improve outcomes was considered to be any intervention (s) provided at a POC, and not the patient's POC visit itself. Therefore, we investigated patients who visited the POC over the same period of time, and compared postoperative outcomes between patients who received any intervention (s) at the POC and those who did not, to explore effects of the POC intervention (s) on outcomes.

### Roles of the POC

The roles of a multidisciplinary POC include early assessments of patients' preoperative conditions allowing for early recognition of needs for additional examinations and specialty consultations, optimization of airway conditions to prevent airway injuries and postoperative infections, optimization of medication management, and enhancement of close, mutual communications among medical providers, patients, and their families^1, 16)^. ASA practice advisories suggest POC benefits from a variety of interventions^17)^. However, only a few studies report beneficial effects of the POC on clinical outcomes, mostly by exploring impacts of interventions provided by a single profession alone^5-11)^.

### Roles of dentists and dental hygienists

In this study, although the dental staff provided interventions in 73.4% of patients receiving POC intervention (s), frequencies of postoperative infections did not differ between the groups. Because our study included various surgical procedures, we could not detect beneficial effects of oral care on postoperative infections possibly by overlooking such effects in patients undergoing high-risk surgery.

In this study, however, frequencies of airway injury did not differ between intervention and non- intervention groups, and further, tooth injuries occurred in neither group. These results suggested that preoperative dental interventions, such as mouth guard preparation and/or oral cleaning, could have, at least, reduced frequencies of tooth injuries in patients in the intervention group with fragile teeth and/or poor oral hygienic conditions to a comparable level seen in patients in the non-intervention group almost free from oral problems.

### Roles of pharmacists

Pharmacists are more likely to prevent medication-associated errors at the POC than in the ward after hospitalization, through earlier and more comprehensive identification of patients' medication requiring appropriate perioperative guidance^18)^. It should be noted that along with anti-coagulants, substantial numbers of CAMs taken by a number of patients require precautions for their perioperative uses^19)^.

### Roles of nurses

Reportedly, roles of preoperative nursing care include preoperative screening and assessment, coordination, communication and collaboration, patient and family education, patient- and family- centered care, preoperative patient contact, and scheduling^20)^. Our study explored ‘collaboration' as an essential task to enhance preoperative management via close, mutual communications among nurses, other medical care providers, patients, and their families.

### Roles of anesthesiologists

Major roles of anesthesiologists at our POC include evaluating patients' health condition as thoroughly as possible, ordering additional examinations depending on patients' comorbidities, and consulting with specialists for expert advice. Another essential role of anesthesiologists at our POC not included in this study is responding to consultations from attending physicians regarding how to manage perioperative care, including anesthesia, in patients with severe comorbidities.

### Clinical outcomes

To date, studies regarding a role of the POC in reducing mortality are limited. Only one study by Blitz et al.^6)^, which compared 35,535 and 28,883 patients visiting and not visiting a POC, respectively, reported a small but statistically significant reduction in mortality in the visitors (0.06% *vs.* 0.08%). Given the low mortality rate, studies enrolling a large number of patients would be required to demonstrate mortality benefits.

Before PS matching in this study, the intervention group included higher proportions of the elderly, females, patients with hypertension, patients with cardiovascular disease, and/or patients classed as ASA-PS ≥ 2, compared with the non-intervention group. Therefore it could be expected that the intervention group would be associated with more frequent and severer postoperative complications. However, no significant inter-group difference was found in frequencies nor severities of postoperative complications. These results suggested that POC intervention(s) could have improved clinical outcomes in the intervention group in such a way that frequencies/severities of postoperative complications in higher-risk patients who required POC intervention(s) could have been reduced to a level comparable to those seen in lower-risk patients mostly not requiring a POC intervention. However, we could not detect such beneficial effects of POC interventions, as no significant difference was found in frequencies or severities of postoperative complications between the PS-matched groups with comparable risk factors. Inclusions of a relatively small number of patients and a wider variety of surgical procedures, compared with previous studies^6, 21-24)^, might have hindered this study from showing beneficial effects of POC interventions. However, the possibility could not be excluded that preoperative dental interventions delivered in 73.4% of patients in the intervention group could have prevented airway injuries, especially tooth injuries, and further, postoperative infections in patients with fragile teeth and/or poor oral hygienic conditions.

### Study limitations

There were some limitations of this study. This study was a retrospective observational study conducted at a single institution including a relatively small number of patients. As this study included patients scheduled for various surgical procedures, different results might have been obtained from studies with different study designs, e.g. focusing on specific surgical procedures. A larger observational study involving more extended periods of time would be required to examine whether POC interventions can exert any beneficial effects on clinical outcomes.

## Conclusion

We introduced our POC consisting of a multidisciplinary team and examined its effects on clinical outcomes. We could not clearly demonstrate beneficial effects of interventions provided by the multidisciplinary POC team on postoperative outcomes, possibly because this retrospective observational study included a relatively small number of patients, multiple types of POC interventions, and various surgical procedures. However, the possibility could not be excluded that early interventions provided by the multidisciplinary POC team might exert some beneficial effect. The impact of POC interventions on clinical outcomes warrants further evaluation.

## Data availability

The datasets related to this study are available from the corresponding author on reasonable request.

## Funding

This work was funded by the Department of Anesthesiology and Pain Medicine, Faculty of Medicine, Juntendo University.

## Author contributions

YU, SI, and EI conceptualized this study. YU, SI, and OK collected data. YU, SI, SN, OK, and MH analyzed data. YU wrote the first draft of the manuscript. SI, GD, EI, and MH edited the manuscript. All authors read and approved the final manuscript.

## Conflict of interest

The authors declare that there are no conflicts of interest.
